# LPS-Induced Galectin-3 Oligomerization Results in Enhancement of Neutrophil Activation

**DOI:** 10.1371/journal.pone.0026004

**Published:** 2011-10-21

**Authors:** Marise Lopes Fermino, Claudia Danella Polli, Karina Alves Toledo, Fu-Tong Liu, Dan K. Hsu, Maria Cristina Roque-Barreira, Gabriela Pereira-da-Silva, Emerson Soares Bernardes, Lise Halbwachs-Mecarelli

**Affiliations:** 1 Université Paris Descartes, Sorbonne Paris Cité, Paris, France; 2 Department of Cellular and Molecular Biology and Pathogenic Bioagents, Faculty of Medicine, University of São Paulo, Ribeirao Preto, São Paulo, Brazil; 3 Program of Basic and Applied Immunology, Faculty of Medicine, University of São Paulo, Ribeirao Preto, São Paulo, Brazil; 4 Department of Biologic Sciences, University of São Paulo, São Paulo, Brazil; 5 Ribeirao Preto College of Nursing, University of São Paulo, Ribeirao Preto, São Paulo, Brazil; 6 Department of Dermatology, School of Medicine, University of California Davis, Sacramento, California, United States of America; 7 Institute of Molecular Pathology and Immunology, University of Porto, Porto, Portugal; Universite de la Mediterranee, France

## Abstract

Galectin-3 (Gal 3) is a glycan-binding protein that can be secreted by activated macrophages and mast cells at inflammation sites and plays an important role in inflammatory diseases caused by Bacteria and their products, such as lipopolysaccharides (LPS). Although it is well established that Gal 3 can interact with LPS, the pathophysiological importance of LPS/Gal 3 interactions is not fully understood. Data presented herein demonstrate for the first time that the interaction of Gal 3, either via its carbohydrate binding C-terminal domain or via its N-terminal part, with LPS from different bacterial strains, enhances the LPS-mediated neutrophil activation *in vitro*. Gal 3 allowed low LPS concentrations (1 µg/mL without serum, 1 ng/mL with serum) to upregulate CD11b expression and reactive oxygen species (ROS) generation on human neutrophils *in vitro* and drastically enhanced the binding efficiency of LPS to the neutrophil surface. These effects required LPS preincubation with Gal 3, before neutrophil stimulation and involved specific Gal 3/LPS interaction. A C-terminal Gal-3 fragment, which retains the lectin domain but lacks the N-terminal part, was still able to bind both to *Escherichia coli* LPS and to neutrophils, but had lost the ability to enhance neutrophil response to LPS. This result emphasizes the importance of an N-terminus-mediated Gal 3 oligomerization induced by its interaction with LPS. Finally we demonstrated that Balb/C mice were more susceptible to LPS-mediated shock when LPS was pretreated with Gal 3. Altogether, these results suggest that multimeric interactions between Gal 3 oligomers and LPS potentiate its pro-inflammatory effects on neutrophils.

## Introduction

Lipopolysaccharide (LPS) represents a major pathogen-associated molecular pattern (PAMP) from the outer membrane of Gram-negative bacteria and is a potent immune activator closely associated with many infectious and inflammatory diseases [1], [2]. LPS recognition by polymorphonuclear neutrophils is one of the first steps of innate immunity. Neutrophil activation by LPS involves both the GPI-anchored molecule CD14 and the Toll-like receptor-4 (TLR-4), with occasional participation of TLR-2 binding to lipoproteins. This activation results in neutrophil degranulation and the activation of the oxidative burst [3]. Since LPS is a powerful immune activator and may be fatal, the response to LPS must be tightly regulated to maintain the immune response at an appropriate level [4].

Gal 3 is a glycan-binding protein composed of a single carbohydrate-recognition domain (CRD) [5]–[7] and an N-terminal aggregation part responsible for its ability to form oligomers, mainly pentamers [8]–[10]. It is widely distributed in tissues, and is predominantly expressed by innate cells, including mast cells, neutrophils and eosinophils [11]. Gal 3 is up-regulated during inflammation/infection, and *in vivo* experiments using Gal 3-deficient mice emphasized the critical role of this protein in regulating inflammatory responses [12]–[15]. Several reports have described the binding of Gal 3 to neutrophil surface and the regulation of neutrophil functions by this protein [16]–[20]. However, the roles of Gal 3 remain unclear, since Gal 3 was claimed either to favour neutrophil recruitment and activation [12]–[15] or to down-regulate the LPS-induced inflammatory responses [21]. One should point out that Gal 3 ubiquitous subcellular distribution and its multiple extracellular and intracellular biological effects render difficult the interpretation of phenotypes observed with Gal 3^−/−^ mice. Our aim was to clarify the modulation by Gal 3 of LPS-mediated neutrophil activation and to investigate Gal 3 mechanisms of action. Data presented herein demonstrate that exogenous Gal 3 lowers the activation threshold of LPS-stimulated neutrophils by interacting directly with LPS and by enhancing its ability to bind neutrophils. Furthermore, the complex formed between LPS and Gal 3 modifies LPS structure, as deduced from fluorescence dequenching and decreases mice resistance to the lethal effects of LPS.

## Methods

### Reagents

Hanks' balanced salt solution with or without Ca^2+^ and Mg^2+^ (HBSS^2+^ and HBSS^2−^) was purchased from Gibco (Paisley, Scotland) and Polymorphprep from Nycomed (Oslo, Norway). LPS from *Klebsiella pneumonia, Escherichia coli* O55:B5, *Escherichia coli* fluorescein isothiocyanate (FITC)-conjugated LPS (FITC-LPS), lactose, sucrose and formyl-Met-Leu-Phe-OH (fMLP) were obtained from Sigma-Aldrich (St. Louis, MO), while LPS from *Salmonella Minnesota R7* was kindly donated by Dr. Catherine Fitting (Pasteur Institute, Paris, FR). LPS preparations were sonicated to prepare homogeneous solutions. Recombinant human tumor necrosis factor alpha (TNF-α) and interleukin-8 (IL-8) were from PeproTech (Rocky Hill, NJ). Desalting Columns were from Thermo Scientific (MA, USA), Detoxi-Gel™ from Pierce (Rockford, IL), anti-CD11b (FITC), -CD14 (FITC), -TLR4 (biotin) monoclonal antibodies, Streptavidin-PE and control isotypes were from AbD Serotec (Düsseldorf, Germany). The polyclonal anti-Gal 3 antibody was produced as previously described [10].

### Galectin-3 preparation

Recombinant human Gal 3 was produced in *Escherichia coli* and purified by as previously described [22]. The lectin was stored at 4°C in phosphate-buffered saline (PBS; pH 7.2) containing 150 mM lactose. Prior to use, lactose was removed by gel-filtration chromatography on desalting columns and galectin 3 was further purified by affinity chromatography on detoxi-gel beads to eliminate contaminating LPS. The carboxyl-terminal domain fragment of Gal 3 (Gal 3C) was prepared by digesting recombinant lectin with collagenase as described [22]. Labeling of Gal 3 with fluorescein isothiocyanate (FITC) was performed essentially as previously described [23].

### Neutrophil preparation

Neutrophils were prepared at room temperature from EDTA-anticoagulated blood obtained at the French Blood Transfusion Center Établissement Français du Sang, with agreement for studies on healthy volunteers. An initial centrifugation for 20 minutes at 120 *g* allowed to recover the platelet-rich plasma, which was further centrifuged separately 10 minutes at 1000 *g* to remove platelets. Blood cells were resuspended in the platelet-poor plasma and neutrophils isolated by one step density-gradient centrifugation on Polymorphprep according to the manufacturer's instructions. Residual erythrocytes were lysed in 0.2% NaCl for 1 minute and the osmolarity of the medium then equilibrated by the addition of an equal volume of 1.6% NaCl. Cells were washed in PBS and resuspended in HBSS^2+^ to a final concentration of 2×10^6^ cells/mL.

### Neutrophil activation and flow cytometry

Neutrophils (5×10^5^) in 250 µl of HBSS^2+^, in 1% BSA-coated tubes, were incubated for 20–45 minutes at 37°C with the indicated molar concentration of Gal 3, LPS, 10^−6^ M fMLP, 25 ng/mL IL-8, 10 ng/mL TNF-α or only HBSS^2+^. Alternatively, neutrophils were stimulated with a mixture containing Gal 3 and LPS. In this case, Gal 3 and LPS were preincubated together at concentrations at least 5 times higher than that used to stimulate neutrophils. After 20 min at 37°C, the Gal 3/LPS mixture was diluted with HBSS^2+^, adjusted to indicated final concentrations and added to the neutrophil pellets. Resuspended neutrophils were then incubated at 37°C for 30 min. For sugar inhibition assays, Gal 3 was pre-incubated with 20 mM lactose or sucrose 15 minutes before use. To evaluate Gal 3 effects on LPS-mediated neutrophil activation in the presence of the LPS binding protein (LBP), 0,2% autologous serum was added to each stimulus condition. For flow cytometry analysis, following the activation, cells were washed with ice-cold PBS, 1% BSA, 0.1% sodium azide (PBA buffer) and incubated for 20 minutes with heat-aggregated goat IgG (1 mg/ml) to block Fcγ receptors, at 4°C, the low temperature and the presence of sodium azide preventing FcγR redistribution and activation. Cells were then treated at 4°C with FITC-labeled anti-CD11b, anti-CD14, or anti-TLR4 mAbs or a control FITC-labeled mouse IgG1 and analyzed by flow cytometry on a Becton Dickinson FACSCalibur (Mountainview, CA).

### Reactive oxygen species generation

PMN (1×10^6^/ml in HBSS^++^) were incubated with the fluorescent probe 2′-7′-dichlorofluorescin hydrodiacetate DCFHDA (5 µM) (Sigma-Aldrich) in BSA-coated tubes at 37°C for 30 min in the dark, with Gal 3 or LPS then with fMLP 10^−6^ M for 7 minutes. The reaction was stopped with ice-cold PBS-azide 0.1%. After centrifugation, the oxydation-dependent fluorescence of intracellular DCFHDA was measured by flow cytometry.

### LPS and galectin-3 binding assay

A flow cytometric assay was developed to assess the effect of Gal 3 on the level of LPS binding on neutrophils. Cells (1×10^5^) in 50 µl of HBSS^2+^ were stimulated for 20 min at 37°C with FITC-labeled *Escherichia coli* LPS (12.5 µg/mL), which had been preincubated with different concentration of Gal 3 (from 0 to 8 µM) or with 4 µM of the truncated form of Gal 3 (Gal 3C). For sugar inhibition assays, lactose or sucrose (12.5 mM) were added to Gal 3, 15 minutes before use. For Gal 3 binding assay, non-activated neutrophils were washed with iced-PBA buffer and treated with 0.2 µM FITC-labeled Gal 3 in presence or absence of 10 mM lactose or sucrose. Following incubations, cells were washed with PBS and the binding of Gal 3 or LPS was measured by flow cytometry.

### Competition assay

Each well in Microtiter plates with U-wells (Nunc, Roskilde, Denmark) was treated with 1 µg of recombinant Gal 3 overnight at 4°C. Plates were rinsed once with PBS, and the unbound protein binding sites in the wells were blocked with 3% (w/v) gelatin in PBS for 2 h at room temperature, followed by three PBS rinses. To each well was added 0.2 µM biotin-labeled Gal 3 and crescent concentration of unlabeled Gal 3 prepared in 1% gelatin, in presence or absence of 5 µg/ml *E.coli* LPS. Plates were incubated for 2 h at 37°C on an orbital shaker and washed with PBS five times. Following incubation with streptavidin-peroxidase for 1 h at 37°C, the Sigma FAST *o*-phenylenediamine dihydrochloride (OPD) substrate was used for development of peroxidase reaction and the optical density was measured at 450 nm.

### FITC-LPS gel filtration and fluorescence analysis

To study the effects of Gal 3 on LPS aggregate structure, 5 µg of FITC-LPS diluted in 200 µL PBS was incubated with 20 µM of Gal 3 or 2% human serum for 2 hours at 37°C. After incubation, the mixture was loaded onto a Superdex TM 75 HR 10/30 column (Pahrmacia, Uppsala, Sweeden), running at a flow of 1 mL/min in PBS. FITC-LPS, Gal 3 or 2% serum alone were also analyzed. Samples were collected into 500 µL fractions and applied in a white 96-wells flat-bottom microtiter plate (Nunc, Roskilde, Denmark), and thereafter the fluorescence was measured in a Microplate Fluorescence reader FLX800 (Bio-Tek Instruments, VT, USA). The excitation and emission wavelengths were 475 nm and 520 nm, respectively.

### Mouse model of endotoxin-mediated mortality

Female Balb/C mice, in groups of five, were challenged with *E. coli* LPS in three different doses (6.2, 12.5 and 25 mg/Kg) diluted in 0.5 mL of PBS, by intraperitoneal (IP) inoculation. Mice were observed for survival for 30 h. Using our established LD_50_ (Lethal Dose, 50%) of LPS, the effect of Gal 3 on LPS**-**induced mortality was assessed by preincubating Gal 3 (40 µM) and LPS (6.2 mg/Kg) in 0.5 mL of PBS for 30 min before IP administration. Control animals received PBS, Gal 3 only or LPS only. The Ethics Committee on Animal Research of the University of Sao Paulo approved all the procedures performed in the study described here.

### Statistical Analysis

The results are expressed as the mean ±SD of the indicated number of animals or experiments. Statistical analysis was performed using analysis of variance followed by the parametric Tukey-Kramer test (Prism 5 GraphPad software, La Jolla, CA, USA). Log rank (Mantel-Cox) test was used to compare the survival rates between the study groups and generate the P value. Differences were considered statistically significant when p≤0.05.

## Results

### Recombinant galectin 3 exerts LPS-dependent and LPS-independent effects on human neutrophils

**Figure 1:**
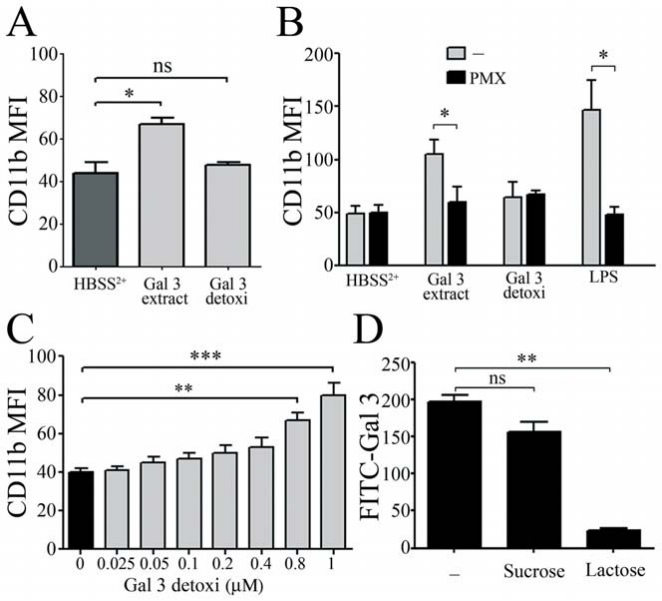
Contaminating LPS interferes with the ability of Gal 3 to activate neutrophils. Freshly isolated neutrophils (2.0×10^6^/ml) were incubated for 45 min at 37°C in HBSS^2+^ in the presence of: **A**: Recombinant Gal 3 preparation (0.2 µM), which had been depleted of endotoxin (detoxi) or not (extract), by absorption on detoxigel (n = 3). **B**: 0.2 µM of extract or endotoxin-depleted recombinant Gal 3, which had been preincubated for 30 min at 37°C with polymyxin B (PMX, 40 mg/ml) (n = 3). **C**: Increasing molar concentrations of endotoxin-depleted Gal 3 (n = 3). After staining with a specific anti-CD11b mAb, cells were analyzed by flow cytometry. **D**: Non-stimulated PMN (2.0×10^6^/mL) were incubated with FITC-labeled Gal 3 (0.2 µM) for 30 min at 4°C, in the presence or absence of 20 mM sucrose or lactose. The binding of FITC-Gal 3 to neutrophils was analyzed by flow cytometry (n = 3). Results are expressed as mean fluorescence intensity (MFI). * p<0.05; **p<0.01; ***p<0.001.

We first analyzed the effects of recombinant Gal 3, isolated from transfected *E. coli* as described [22], without (Gal 3 extract) and with (detoxi) LPS elimination on detoxi-gel and incubated with freshly isolated unprimed neutrophils. As shown in figure 1A, the incubation with 0.2 µM Gal 3 extract resulted in neutrophil activation, as measured by the increase of CD11b expression. This effect was not observed with the same molar concentration of endotoxin-free detoxigel-treated Gal 3 (detoxi-Gal 3). LPS contamination of the Gal 3 extract preparation, as measured by Limulus Amebocyte Lysate test, was less than 400 nanograms of LPS per mg of Gal 3 (data not shown). We then pretreated the Gal 3 extract preparation with polymyxin B (PMX), to block the effect of contaminating LPS. As shown in figure 1B, PMX prevented CD11b up-regulation promoted by Gal 3 extract preparation or by LPS. We then analyzed the effect of increasing doses of endotoxin-free detoxigel-treated Gal 3 on unprimed neutrophils. As shown in figure 1C, detoxi-Gal 3 concentrations up to 0.4 µM had no significant effect on neutrophil activation state. One should point out, however, that high molar concentrations (≥0.8 µM) resulted in a significant up-regulation of CD11b membrane expression which, in this case, was PMX-insensitive.

The binding of Gal-3 to neutrophils surface was investigated, using FITC-labeled detoxi Gal 3. Figure 1D shows that concentrations as low as 0.2 µM detoxi-Gal 3 efficiently bound to neutrophils, and that this binding was specifically inhibited by lactose, thus involving Gal 3 carbohydrate recognition domain. The concentrations of 0.2–0.4 µM Gal 3, allowing this binding of Gal3 without modification of neutrophil CD11b levels, were chosen to analyze the effect of Gal 3 on LPS-induced cell activation.

### Galectin 3 endows low doses of LPS with the ability to activate neutrophils

**Figure 2:**
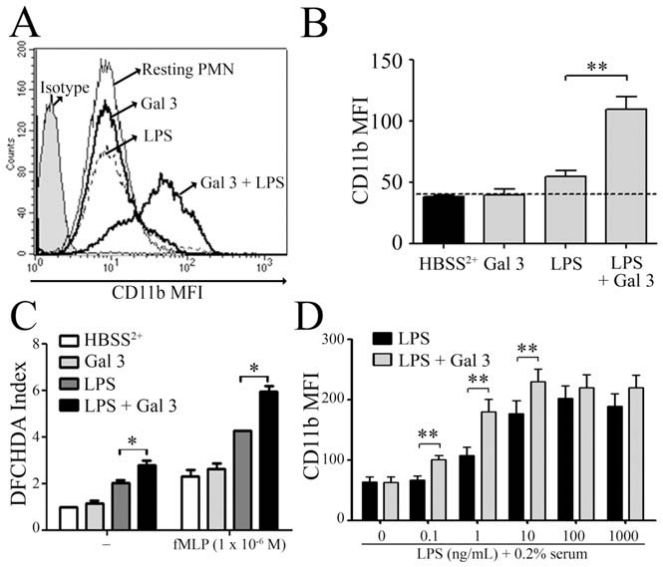
Gal 3 increases the ability of LPS to activate neutrophils and induces oxidative responses. PMNs (2.0×10^6^/ml) were incubated for 45 min at 37°C with *E. coli* LPS (1 µg/mL) and Gal 3 (0.4 µM), which had been preincubated separately or together for 30 min at 37°C; after labeling with a specific anti-CD11b mAb, cells were analyzed by flow cytometry. **A**: Representative histrogram of neutrophil CD11b expression. **B**: Bar graph representation of data obtained from 5 experiments (mean ± SD). **C**: PMNs were incubated at 37°C for 30 min with DCFHDA and either Gal 3 (5 µg/ml), LPS (1 µg/ml) or a mixture of Gal 3 and LPS pre-incubated for 20 min at 37°C. Then, PMN were further activated or not with fMLP 10^−6^ M for 7 minutes. The reaction was stopped by the addition of ice-cold PBS-azide 0.1% and the oxydation-dependent fluorescence of intracellular DCFHDA was measured as described in Methods. The results are given as DCFHDA index, i.e. the mean fluorescence intensity MFI of each sample/MFI of resting PMN (n = 3). **D**: Increasing concentrations of LPS (0.1 to 1000 ng/ml), in 0.2% autologous serum, were preincubated or not with Gal 3 (0.4 µM) and then added to PMNs. CD11b expression was measured as above after a 45-min incubation and expressed as mean MFI ± SD (n = 3). *p<0.05, ** p<0.01.

The effects of Gal 3 on LPS-mediated neutrophil activation were then investigated. LPS is known to lose most of its neutrophil activating efficiency in the absence of serum LPS-binding protein (LBP) and soluble CD14 [24] and concentrations as high as 10 µg/ml of LPS are required to activate neutrophils in the absence of serum (data not shown). When unprimed human neutrophils were incubated with suboptimal *E. coli* LPS concentration (1 µg/ml) or with 0.4 µM detoxi-Gal 3, separately, these stimuli induced little or no neutrophil activation (Figure 2A). By contrast, a two- to three-fold increase of CD11b expression was observed when neutrophils were treated with the same amount of LPS that had been preincubated with Gal 3 (Figure 2A, 2B). We also tested whether Gal 3 was able to enhance neutrophil oxidative responses to LPS. LPS is not an efficient trigger of the oxidative burst but it is known to prime PMN for an enhanced oxidative response to fMLP [25]. Therefore, we first primed PMN with either Gal 3, LPS or a pre-incubated mixture of Gal 3 and LPS, in the presence of the fluorescent probe DCFHDA, and then further activated PMN with fMLP. The results show that LPS preincubation with Gal 3 increases the oxidant response of PMN to LPS itself (DCFHDA indexes of 2.77±0,2 and 2±0.09 with LPS/Gal3 and LPS alone, respectively) (Figure 2C). Moreover, it potentiates LPS priming effect: PMN preincubation with the LPS/Gal3 mixture lead to up to 3 fold the oxidative response to fMLP, as compared to the 1.8 fold increase observed after priming by LPS alone (Figure 2C).

Finally, the effects of Gal 3 on neutrophil activation were also tested with LPS in the presence of serum factors such as LBP and CD14. In the presence of 0.2% normal human serum, as low as 1 ng/mL of LPS were able to activate neutrophils. Here again, Gal 3 caused a further decrease in the LPS threshold since, in the presence of serum, 0.1 ng/mL LPS preincubated with Gal 3 induced an increase of neutrophil CD11b expression similar to 1 ng/mL LPS alone (Figure 2D).

### Galectin-3 effects on LPS-mediated activation require galectin-3/LPS interactions

**Figure 3:**
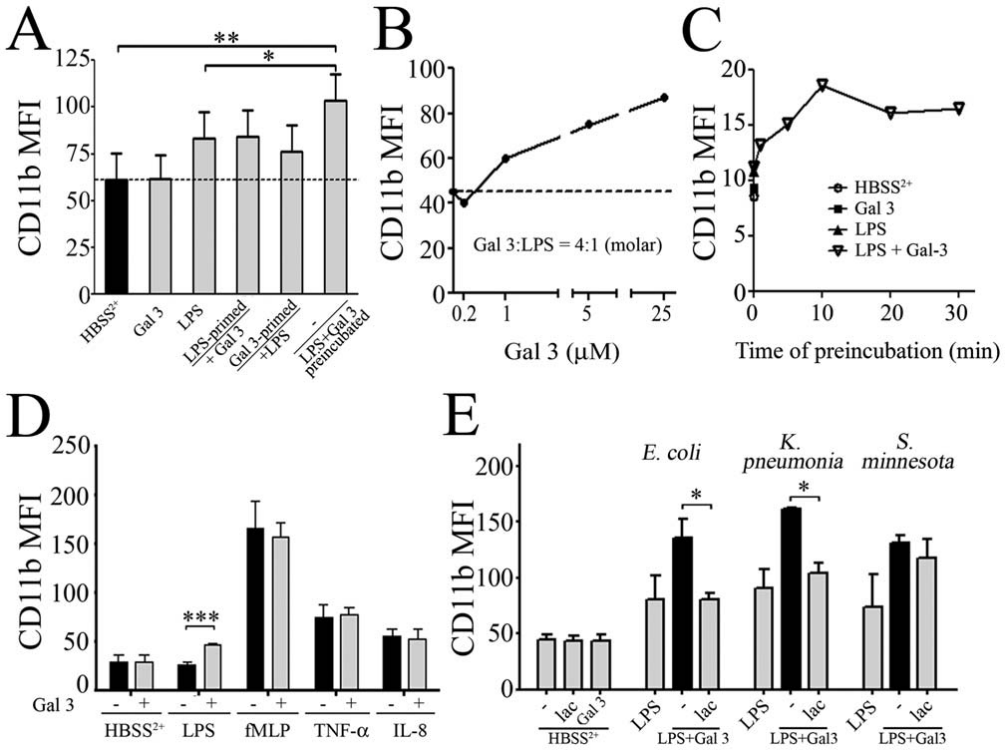
Gal 3 specifically enhances LPS-mediated neutrophil activation and requires a direct interaction with LPS for its effect. **A**: PMNs were primed for 30 min at 37°C with Gal 3 (0.2 µM) or *E. coli* LPS (1 µg/mL) and then stimulated with LPS (1 µg/mL) or Gal 3 (0.2 µM), respectively. Alternatively, Gal 3 (1 µM) and LPS (5 µg/mL) were preincubated together for 30 min at 37°C and then diluted to 0.2 µM (Gal 3) and 1 µg/mL (LPS) to be used to stimulate PMNs (n = 2). **B**: Effect of increasing LPS and Gal 3 concentrations in the pre-incubation mixture. Increasing concentrations of Gal 3 (0.2 µM to 25 µM) and LPS (1 µg/mL to 125 µg/mL) were preincubated together for 30 min at 37°C. They were then diluted in HBSS^2+^ to the final Gal 3 − 0.2 µM and LPS – 1 µg/ml concentrations and added to PMN for a 45-min incubation at 37°C. **C**: Time-response curve of LPS and Gal 3 preincubation. Gal 3 (7 µM) and LPS (35 µg/mL) were preincubated together for 0–30 min, as indicated, at 37°C and then diluted to 0.2 µM (Gal 3) and 1 µg/mL (LPS) to be used to stimulate PMNs for a 45-min incubation at 37°C (representative from 2 independent experiments). **D**: PMNs were stimulated with LPS (1 µg/ml), fMLP (10^−6^ M), TNF-α (10 ng/ml) or IL-8 (25 ng/ml), which had all been preincubated without (black) or with Gal 3 (0.4 µM, grey) for 30 min at 37°C. Neutrophil CD11b expression was measured as in figure 1 (n = 5). **E**: Gal 3 effect on neutrophil activation by LPS from various strains of bacteria. Gal 3 (1 µM) was preincubated with 10 µg/ml LPS from *E. coli*, *K. pneumonia* or *S. minnesota R7* for 30 min at 37°C, in presence or absence of 10 mM lactose (lac). Then, each mixture was diluted to 0.2 µM (Gal 3) and 1 µg/mL (LPS) concentrations and used to stimulate PMNs (2×10^6^/mL) for 45 min at 37°C, and analyzed for CD11b expression as above. Results are expressed as mean fluorescence intensity (MFI) (n = 3). * p<0.05, **p<0.01, ***p<0.001.

We initially concluded, as others [26], [27], that the effect of Gal 3 on neutrophils required cell priming by LPS. However, surprisingly, the enhancing effect of Gal 3 on LPS-mediated neutrophil activation was not observed when neutrophils were primed with 1 µg/mL of LPS before the addition of Gal 3 (0.2 µM) or vice-versa (Figure 3A). Indeed, LPS- or Gal 3-primed cells did not differ significantly from cells treated with Gal 3 or LPS alone (Figure 3A). Importantly, Gal 3 effects on neutrophil activation required a prior interaction of Gal 3 with LPS, since only the pre-incubated Gal 3/LPS was able to significantly increase neutrophil activation (Figure 3A). In fact, we observed a dose-dependent up-regulation of CD11b expression with increasing concentrations of LPS/Gal 3 during the preincubation step, although the final concentration in contact with neutrophils was kept constant (0.2 µM Gal 3, 1 µg/ml LPS) (Figure 3B). Moreover, a time-response curve for LPS/Gal 3 preincubation revealed that the ability of Gal 3 to interact with LPS and to induce CD11b up-regulation of expression was very rapid in onset, reaching a plateau after 10 min of preincubation (Figure 3C). These results further emphasize the importance of a direct interaction of Gal 3 with LPS. The ability of Gal 3 to enhance neutrophil activation was specifically observed with LPS and not upon pre-incubation with other stimuli such as fMLP, TNF-α or IL-8 (Figure 3D).

### Galectin-3/LPS-mediated neutrophil activation relies on N- or C-terminal parts of Gal 3, according to LPS bacterial strains

In order to investigate the Gal 3 parts involved in the observed effects, we analyzed LPS from different strains known to interact either with gal 3 lectin domain (*Klebsiella pneumonia*), with gal 3 N-terminal part (*Salmonella minnesota R7* (Rd mutant) or with both N-terminal and lectin parts (*E. coli*) [21], [28]. As shown in Figure 3E, Gal 3 endows all three strains of LPS with an enhanced ability to up-regulate neutrophil CD11b (black bars). This effect was inhibited by lactose in the case of *E. coli* LPS and *K. pneumonia,* but not in the case of *S. minnesota* R7 LPS, confirming that the lectin domain is not involved in Gal 3 interaction with this latter LPS. Altogether, these results indicate that Gal 3 effects on LPS-mediated neutrophil activation require an interaction of LPS with either the glycan-binding or the N-terminal part of Gal 3.

### Gal-3 strikingly enhances the interaction of LPS with neutrophils

**Figure 4:**
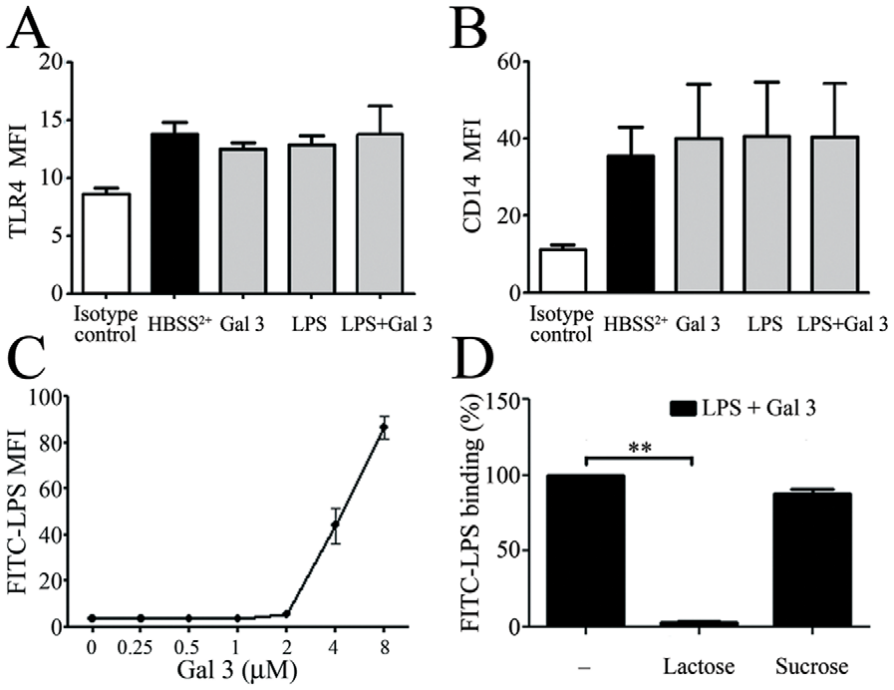
Gal 3 enhances LPS binding to the neutrophil surface. **A and B**: PMNs (2×10^6^/mL) were stimulated for 45 min with Gal 3 (0.4 µM) and *E. coli* LPS (1 µg/ml), preincubated as in figure 3. TLR4 (**A**) and CD14 (**B**) expressions were determined by flow cytometry. The results are expressed as mean fluorescence intensity (MFI) (n = 3). **C**: FITC-labeled *E. coli* LPS (12.5 µg/mL) was preincubated with increasing molar concentrations of Gal 3 (from 0.25 to 8 µM) and then added to PMNs (10×10^6^/mL) for additional 20 min incubation at 37°C with (n = 3) **D**. Gal 3 was treated with 12.5 mM lactose or sucrose for 15 min before being incubated with FITC-LPS (12.5 µg/mL) and added to PMNs (10×10^6^/mL). The binding of FITC-LPS was determined by flow cytometry and expressed as mean fluorescence intensity (MFI).

To test whether the Gal 3-mediated increase in LPS binding might be caused by an up-regulation of LPS receptors on neutrophils, we analyzed the expression of CD14 and TLR4. As shown in figure 4, there was no significant change in TLR4 (A) and CD14 (B) expressions after neutrophil stimulation with the Gal 3/LPS complex. We then hypothesized that Gal 3 enhances the effects of LPS by increasing its binding to neutrophil surface. We thus preincubated FITC-labeled *E. coli* LPS with increasing concentrations of Gal 3 for 30 min before adding it to neutrophils. One should point out that low concentrations of FITC-LPS (1 µg/mL) preincubated with 0.4 µM of Gal 3, in the absence of serum, did not result in a level of fluorescence intensity detected by flow cytometry. Therefore, we increased proportionally the doses of FITC-LPS and Gal 3 and found that the dose of 12.5 µg/mL LPS allowed the detection of cell-bound LPS. Preincubation of this amount of LPS with increasing concentrations of Gal 3 showed a significant increase of LPS binding to neutrophils for a Gal 3/LPS proportion (4 µM Gal 3 per 10 µg LPS) similar to that required for neutrophil activation. As expected, previous treatment of Gal 3 with lactose (12.5 mM) specifically inhibited the Gal 3-induced binding of LPS to neutrophils (Figure 4D). Gal 3/LPS interactions thus strikingly enhance the binding of LPS to the cell surface.

### LPS induces N-terminal part-dependent Gal 3 oligomerization

**Figure 5:**
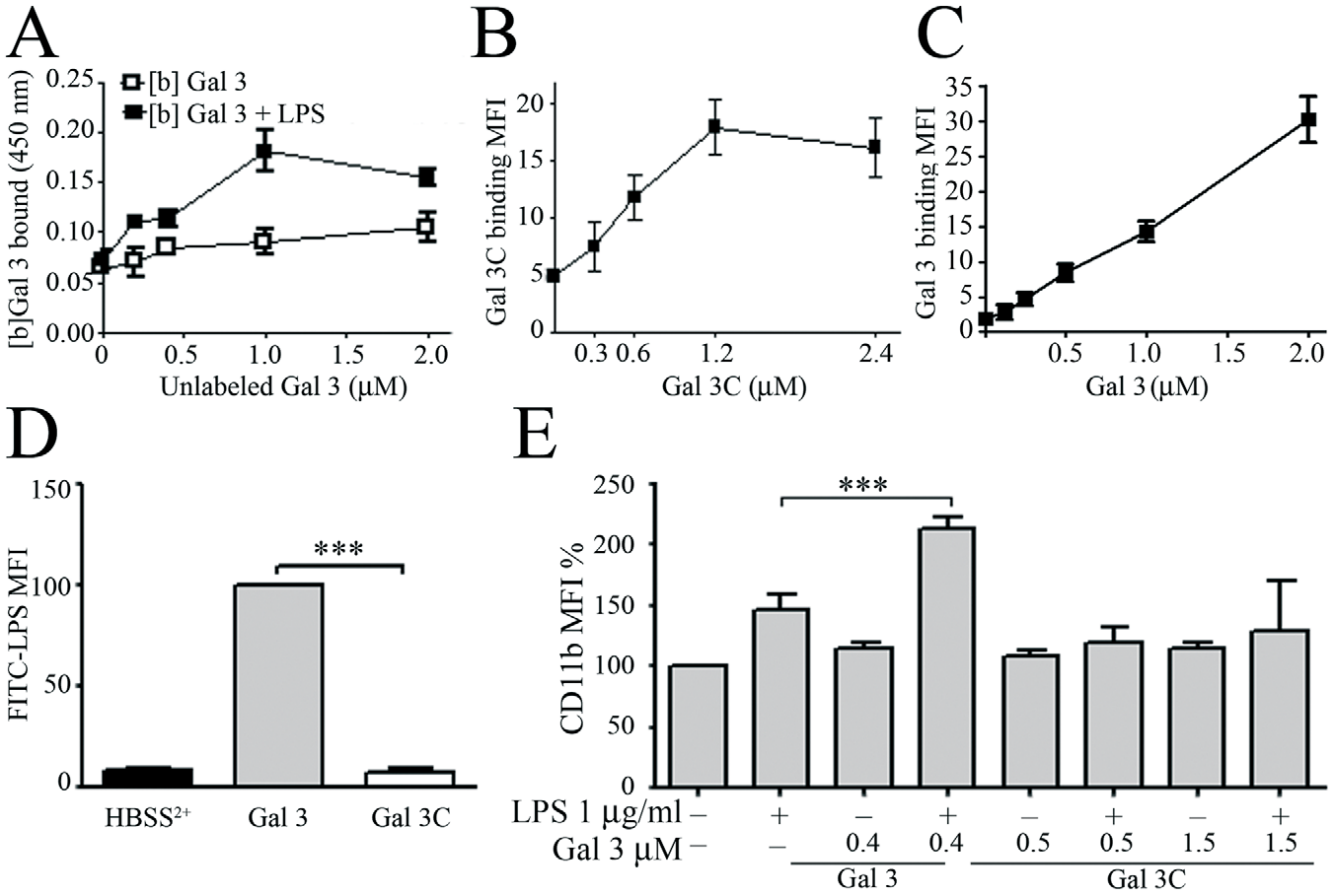
LPS induces oligomerization of Gal 3. **A**: A 96-well microplate was coated with 1 µg of recombinant Gal 3 per well. After blocking with gelatin 3%, 0.2 µM biotin-labeled Gal 3 ([b]Gal 3) and the indicated concentrations of unlabeled Gal 3 were added to each well in the presence (solid squares) or absence (open squares) of 5 µg/mL *E. coli* LPS. The signal was generated by neutravidin-HRP using OPD as a substrate. **B and C**: PMNs (2×10^6^/mL) were incubated for 30 min at 4°C with increasing concentrations of truncated Galectin-3 (Gal 3C), lacking the N-terminal part (**B**), or increasing concentration of full-length Galectin-3 (Gal 3) (**C**). After washing, a specific polyclonal goat anti-Gal 3C (**B**) or goat anti-Gal 3 (**C**) and an FITC-labeled secondary anti-goat IgG antibody were used to detect Gal 3C on neutrophils by flow cytometry (n = 2). **D**: PMNs (10×10^6^/mL) were treated for 20 min at 37°C with FITC-LPS (12.5 µg/mL) preincubated with Gal 3 (8 µM) or with Gal 3C (8 µM). The binding of FITC-LPS was evaluated by flow cytometry as in figure 4. **E**: PMNs (2×10^6^/mL) were treated for 45 min at 37°C with the indicated concentrations of full-length Gal 3 (Gal 3) or Gal 3C, preincubated or not with *E. coli* LPS (1 µg/mL), and analyzed for CD11b expression by flow cytometry. ***p<0.001.

Since the binding of FITC-LPS to neutrophils is more pronounced as the concentration of Gal 3 reaches a threshold concentration (2 µM, Figure 4C), we next tested the ability of LPS to trigger galectin-3 self-association. Biotin-labeled Gal 3, without or with LPS, was allowed to interact with a galectin 3-coated plate, in the presence of increasing amounts of unlabeled Gal 3. As shown in figure 5A, the unlabeled Gal 3 did not compete with the biotin-galectin 3, since the amount of biotin-galectin 3 binding was not decreased, but on the contrary slightly enhanced in the presence of a 10 time excess of unlabeled Gal 3. This suggests the occurrence of Gal 3 oligomerization, which was enhanced in the presence of LPS (Figure 5A).

In order to test the hypothesis that LPS induces Gal 3 oligomerization via the N-terminal part of the molecule, we used the C-terminal fragment Gal 3C, generated by collagenase digestion of Gal 3, which lacks the N-terminal part but still possesses the carbohydrate-binding activity. First, Gal 3C was shown to be able to bind to neutrophils, as detected with a polyclonal anti-Gal 3C antibody (Figure 5B). This binding was saturable, reaching a plateau at about 1.2 µM Gal 3C, unlike that observed with FITC-labeled full-length Gal 3 (Figure 5C). This non-saturable binding illustrates the ability of the full-length Gal 3, at high concentration, to form multimers, via its N-terminal part, on neutrophils, while Gal 3C had lost this ability. Similarly, contrasting with the full-length Gal 3, Gal 3C was unable to increase the binding of FITC-LPS to neutrophils (Figure 5D) and to enhance the LPS-mediated neutrophil activation, as shown in Figure 5E, where LPS was preincubated with Gal 3C concentrations up to four times greater than those used for full-length Gal 3. These results emphasize the importance of N-terminal part-mediated Gal 3 oligomerization in its ability to enhance LPS binding to and activation of neutrophils.

### Gal 3/LPS interaction leads to an increase in the fluorescence of FITC-LPS in a similar way as serum LBP protein

**Figure 6:**
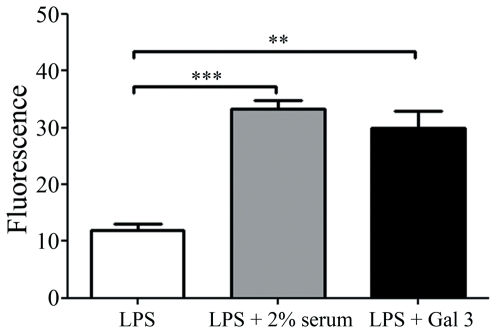
Gal 3/LPS interaction enhances the fluorescence of FITC-LPS. FITC-labeled *E. coli* LPS (10 µg/ml) was incubated with 4 µM Gal 3 or 2% serum for 2 hours, and then applied to a gel filtration column (Superdex TM 75 HR 10/30 column). FITC-LPS alone or Gal 3 alone were also analyzed. 0.5 ml fractions of FITC-LPS ± Gal 3 or ±2% serum were collected and further analyzed on a fluorescence microplate reader. The excitation and emission wavelengths were at 475 nm and 520 nm, respectively. Data represents the fluorescence of the main peak of FITC-LPS recovered in the void volume. ** p<0.01, ***p<0.001.

The fact that Gal 3/LPS interaction induces greater-than 20-fold increase in FITC-LPS binding to neutrophils but only a three-fold increase in LPS-mediated CD11b up-regulation raised the possibility that galectin-3 might interfere with the intramolecular self-quenching of fluorescein. It has long been known that the fluorescence of FITC-LPS aggregates is self quenched but should increase when the aggregates dissociate [29] or the spacing between FITC-LPS molecules within aggregates increase [30], because of dequenching. Therefore, FITC-LPS, preincubated or not with Gal 3 or with serum LPB, was analyzed by gel filtration followed by fluorescence detection. Figure 6 represents the analysis of the main peak of FITC-LPS recovered in the void volume and clearly demonstrates that the presence of Gal 3 enhances three times the FITC-LPS fluorescence. Similar results were obtained when FITC-LPS was preincubated with serum LBP, which it is known to promote LPS disaggregation [31]. This result suggests that Gal 3/LPS interaction is able to modify the LPS aggregate structure, which may account, at least in part, for its potentiating effect on LPS-induced neutrophil activation.

### Exogenous Gal 3 increases mortality to endotoxin shock induced by LPS

**Figure 7:**
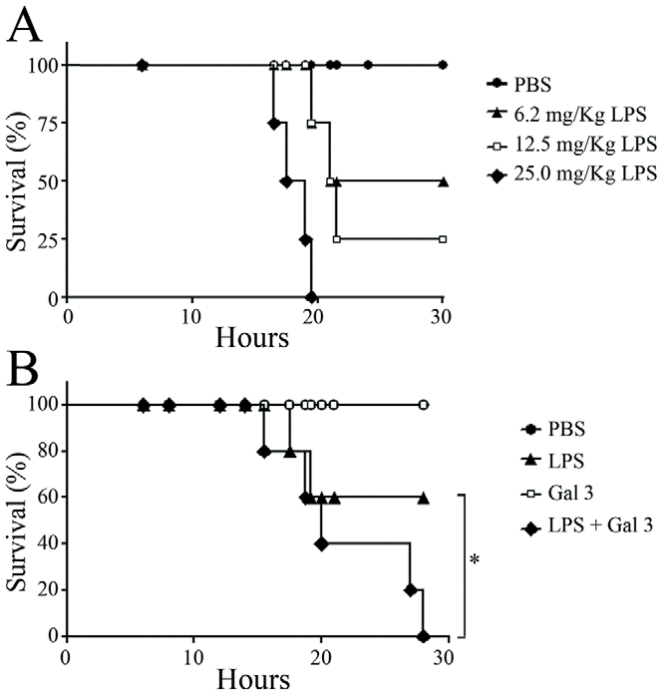
Gal 3/LPS interaction decreases mice resistance to the lethality of LPS. **A**: Groups of 5 Balb/C mice were challenged with PBS, 6.2, 12.5 or 25 mg/kg of *E. coli* LPS by intraperitoneal (ip) injection. **B**: Mice (5 per group) were challenged with 500 µl of PBS, Gal 3 (40 µM), 6.2 mg/kg LPS or with a mixture of LPS (6.2 mg/kg) and Gal 3 (40 µM) preincubated for 15 min. The mortality rate was monitored regularly and represented as percentage of survival. *p<0.05.

Endotoxic shock is the most severe form of LPS-mediated inflammatory disease, which is mainly mediated by an uncontrolled neutrophil activation [32]–[36]. So far we have shown that Gal 3 lowers the activation threshold of LPS-stimulated neutrophils *in vitro*. We, thus, further explored Gal 3 effect in an *in vivo* sepsis model. First, in order to determine a suitable concentration of LPS for inducing endotoxemia, mice were challenged with different doses of LPS (Figure 7A). All mice receiving 25 mg/Kg LPS died, while those receiving LPS at 6.2 mg/Kg exhibited 50% survival during a 30-hour period. Therefore, we assessed the influence of Gal 3 on endotoxin-mediated mortality by measuring survival of mice challenged with LPS (6.2 mg/Kg) preincubated with 500 µL of Gal 3 (40 µM) for 15 minutes before the challenge. As shown in figure 7B, all mice receiving PBS or Gal 3 alone survived, while 60% of mice receiving LPS at 6.2 mg/Kg survived up to 30 h past challenge, as expected. Interestingly, all mice challenged with LPS pre-incubated with Gal 3 died within 30 h after challenge. This result suggests that exogenous Gal 3 can enhance the effect of LPS both *in vitro* and *in vivo*.

## Discussion

The major conclusion of this study is that galectin-3 decreases ten times the LPS concentration threshold for neutrophil activation. This may be important in inflammation sites where Gal 3, secreted in situ by macrophages or mast cells, may render neutrophils particularly sensitive to LPS.

Various reports have described Gal 3 in vitro pro-inflammatory effects on neutrophils, which required cell priming with fMLP [17] or TNF-α [37]. Similarly, Almkvist and colleagues previously stated that neutrophils had to encounter bacterial LPS first, in order to be activated by galectin-3, on the basis of results showing that Gal 3 triggered responses of neutrophils pre-incubated with 10 µg/ml LPS [27]. In our experimental settings, which used LPS concentrations as low as 1 µg/ml in the absence of serum or 1 ng/ml with serum, Gal 3 had no effect on LPS-pretreated neutrophils. However, Gal 3 pre-incubation with LPS enhanced the ability of these low LPS concentrations to activate naïve, unprimed neutrophils, as shown by the upregulation of CD11b expression. Gal 3 specifically potentiated neutrophil activation by LPS and had no effect on cell activation by other agonists such as fMLP, TNF-α or IL-8. Indeed, our results emphasize the importance of specific Gal 3/LPS interactions, as shown by a concentration dependent effect of Gal 3 preincubation with LPS on the resulting neutrophil activation (Figure 3B). The required concentrations for the galectin 3/LPS pre-incubation step are high but are most probably reached when Gal 3 secreting macrophages get in contact with LPS-releasing bacteria in inflamed tissues. Moreover, the final Gal 3 concentrations used to stimulate neutrophils were similar or lower to those used in previously reported in vitro data on Gal 3 effects [16], [17], [19], [20], [26], [27].

Our results confirm that some of the in vitro effects of recombinant *E. coli*-derived Gal 3 preparations are due to an LPS contamination and are inhibited by polymyxin B [38]. The detoxified LPS-free Gal 3 efficiently bound to neutrophils, via its carbohydrate recognition domain but had no significant effect on neutrophils at concentrations lower than 0.8 µM.

Two independent LPS binding sites on galectin-3 have been demonstrated so far. One is the carbohydrate binding site in the C-terminal domain of Galectin-3, which confers binding to β-galactoside-containing LPS from *K. pneumonia*. In contrast, *S. minnesota* LPS is devoid of β-galactosides, but can bind to a site within the N-terminal domain of galectin-3 through its Lipid A part, known to be essential for LPS-mediated bioactivity [28]. In addition, *E. coli* LPS binds to galectin-3 both through Lipid A and β-galactoside-containing polysaccharide chains [21]. The analysis of LPS from different strains of bacteria showed that Gal 3 interaction with *E. coli*, *K. pneumonia* and *S. minnesota* LPS enhanced LPS-mediated neutrophil activation. Accordingly, blocking the lectin domain with lactose inhibited the effect of Gal 3 on *E. coli* and *K. pneumonia* LPS but not its effect on *S. minnesota* LPS, further demonstrating that, no matter what domain is involved, the interaction between LPS and Gal 3 is essential to the enhanced neutrophil activation mediated by LPS.

The effect of Gal 3 was further investigated with Gal 3 C-terminal fragment Gal 3C, which retains its ability to bind, via its lectin site, to neutrophil membrane (Figure 5B). Although this truncated Gal 3 is able to bind *E. coli* LPS, it had lost its ability to enhance the binding of LPS to neutrophils (Figure 5D) and the resulting cell activation (Figure 5E). These results emphasize the importance of Gal 3 oligomerization, via its N-terminal part, in the LPS-enhancing effect.

Gal 3 oligomerization could also account for the striking increase of LPS binding to neutrophils, when LPS was pre-incubated with Gal 3, in spite of a constant number of LPS receptors, TLR4 and CD14.

It is well established that, in the absence of ligands, galectin-3 is monomeric, but it oligomerizes upon binding to synthetic multivalent or cell surface glycans, through self-association of N-terminal parts [8], [22], [39]. Efficient oligomerization, in the presence of ligands such as IgE, was shown to require a minimal galectin-3 concentration of 1.0 µM [22]. Similarly, galectin-3 oligomerization was reported to occur on the surface of neutrophils [18] but with 2 µM Gal 3 concentration, i.e. 10 times that of our experimental setting, which is probably not sufficient to allow the formation of oligomers on the cell surface. The fact that the enhancement of LPS-mediated neutrophil activation was only achieved if galectin-3 had been preincubated, at concentrations equal or higher than 1 µM, with LPS, strongly suggests that LPS itself induces galectin-3 oligomerization during this preincubation step. This was confirmed by the analysis of the formation of galectin 3 oligomers on galectin 3-coated plates, which was significantly enhanced by the presence of LPS (Figure 5A). To our knowledge, this is the first observation of galectin-3 oligomerization promoted by LPS.

**Figure 8:**
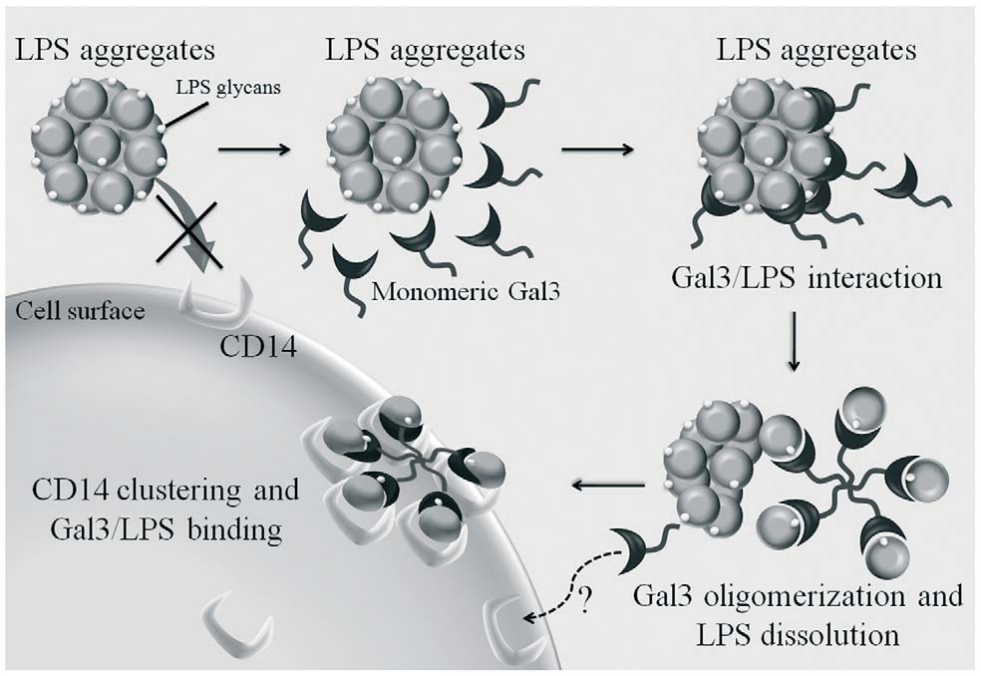
Schematic view of putative mechanisms responsible for galectin-3 enhancement of LPS-induced neutrophil activation. Monomeric Galectin-3 interacts with LPS glycans from LPS aggregates via its C-terminal domain. LPS/Gal 3 interactions induce Gal 3 oligomerization, which promotes the dissolution of LPS aggregates, stabilizes LPS monomers and enhances the LPS interaction with surface receptors, leading to increased neutrophil activation. CD14 only is shown as the initial LPS ligand on neutrophils, because it is highly glycosylated and could, potentially, interact directly with Gal 3. Moreover, being GPI-anchored, CD14 would be most easily clustered on the cell surface. However, this implies a secondary clustering of TLR/MD2, which would transmit or modulate signals into the cell.

Our results raise the question as to how galectin-3 oligomerization could lower the activation threshold of neutrophils to LPS. One possibility is that each galectin-3 oligomer, binding several LPS molecules, cross-links LPS receptors such as CD14 and TLR4 on the cell surface, thereby increasing their cell activating efficiency, as schematized in figure 8. Moreover, simultaneous interaction of galectin-3 carbohydrate recognition domain with glycans on LPS receptors such as CD14 [31], [40], [41] could further enhance the binding of LPS to the cell surface. Another hypothesis, which does not exclude the former one, is that galectin 3 dissociates LPS aggregates, as has been observed with LPS-binding protein (LBP). Indeed, LPS, in aqueous suspension, forms aggregates with low cell activating efficiency, which are disrupted by LPS-binding protein (LBP) [42]. This accelerates LPS binding to CD14 and enhances the resulting TLR-4-mediated cell signaling. We observed that the fluorescence of FITC-LPS was enhanced upon incubation with Galectin 3 (Figure 6), which indicates that galectin-3 somehow interferes with LPS aggregate structure. However, at this point we are not able to assert that Gal 3 promotes LPS disaggregation in a way similar to LBP or that it just interferes with the space between FITC molecules, thus promoting dequenching [30]. Gal 3 putative dissociation of LPS aggregates (Figure 8) could account for the ability of low concentrations of LPS to stimulate neutrophils, when pre-incubated with Gal 3, even in the absence of serum LBP. An intriguing observation is galectin-3 enhancement of cell activation induced by *Salmonella minesota* LPS, which only binds to galectin-3 via its N-terminal part. This indicates that galectin-3 oligomerization may be triggered by the engagement of the N-terminal part with ligands, an observation not described so far, opening a new avenue in the study of galectin-3 functions.

When LPS reaches the systemic circulation, it induces a massive release of proinflammatory cytokines, that cause the pathophysiological state known as endotoxic shock [43]. Our results showing an increased mortality of mice challenged with LPS preincubated with galectin-3 do not proove that the increased mortality is due to the direct activation of neutrophils by LPS/Gal3 complexes. It could be due to LPS/Gal3 interactions with resident cells such as macrophages, resulting in increased secretion of TNF-α, IL-8 and other cytokines and ultimately in neutrophil hyperactivation. These results, however, definitely argue in favour of an amplifying role for galectin-3 in LPS-induced cellular responses. This contrasts with the data of Li and colleagues [21], who demonstrated that gal3^−/−^ mice were more susceptible to endotoxin shock and observed dramatically increased galectin-3 serum levels in wild-type mice 4 h after LPS challenge. The reason for this discrepancy may be searched in the analogy between Gal 3 and LBP behavior towards LPS. Indeed, in spite of the enhancing effect of LBP on LPS signalling at low concentration, LBP inhibits this signalling, when present in high concentration, by shuttling LPS to the serum lipoproteins and by formation of LPS aggregates [44], [45]. It has, therefore, been suggested that LBP may serve as an inhibitor of the excessive response to LPS, which would explain its higher levels found in the serum of septic patients [46]. It is tempting to speculate that, in the experimental setting used by Li and colleagues [21], the systemic galectin-3 concentration found after LPS challenge (∼300 ng/mL) would not be sufficient to enhance LPS-induced activation and therefore may exert an inhibitory role. The same authors showed that the in vitro preincubation of LPS with galectin-3 reduced the responsiveness of bone marrow-derived macrophages (BMMs) to LPS. However, BMMs may react differently than neutrophils and, more importantly, they used galectin-3 doses as low as 5–20 nM, which, in our system, would be without enhancing effect.

In conclusion, regarding the physiological function played by LPS-galectin-3 interactions during infections caused by gram-negative bacteria, we propose that galectin-3 could serve as a sensor to detect small amounts of LPS and allow it to efficiently activate recruited neutrophils. Of note, our recent observation that galectin-3-deficient mice (gal3^−/−^) developed a delayed inflammatory response during the early phase (6–24 hrs) of *Rhodococcus equii* infection [47] argues in favour of a role for galectin-3 as a pathogen danger signal in this model. Moreover, galectin-3 could be an innate immune sensor with a broader specificity than LPS. Indeed, galectin-3 has been shown to bind mycolic acid (MA), the major constituents of Mycobacterium tuberculosis cell envelop [48]. Interestingly, these authors also demonstrated that MA interferes with an initial formation of galectin-3 oligomers on a laminin substratum, although galectin-3 oligomerization on immobilized MA was not assayed. The ability of galectin-3 to bind two hydrophobic ligands such as MA and Lipid A, as in *Salmonella minnesota* R7 LPS, opens the possibility that galectin-3 may also interact with Lipoteichoic acid (LTA), a major constituent of the wall cell of gram-positive bacteria. Interestingly, LPS-binding protein also interacts with LTA from gram-positive bacteria and promotes its interaction with CD14 [49]. The possible interaction between galectin-3 and LTA, or other product from gram-positive bacteria, warrants further investigation.
